# Generalized Solvent Effect on the Fluorescence Performance of Spiropyran for Advanced Quick Response Code Dynamic Anti-Counterfeiting Sensing

**DOI:** 10.3390/ijms26041531

**Published:** 2025-02-12

**Authors:** Junji Xuan, Lingjie Chen, Jintao Tian

**Affiliations:** 1 National Key Laboratory of Marine Corrosion and Protection, Luoyang Ship Material Research Institute, Luoyang 471023, China; 2 School of Materials Science and Engineering, Ocean University of China, Qingdao 266100, China

**Keywords:** spiropyran, fluorescence, solvent effect, Hansen solubility parameters, viscosity, quick response code anti-counterfeiting sensing

## Abstract

Spiropyran has an attractive and mysterious fluorescence switch and dual-color conversion characteristics, as it exhibits both aggregation-caused quenching (ACQ) in solvents and fluorescence enhancement in polymer matrices. The explanation for this phenomenon has always been of great controversy. Hence, the solvent effect on the emission of spiropyran (**SP**) was investigated in 16 solvents. By means of molecular orbital theory and the Jablonski diagram, several special parameters (e.g., Hansen solubility parameters and viscosity) were selected for this analysis, with excellent goodness of fit. Subsequently, the main factors that affected the blue shift, red shift, and luminescence efficiency of the emission of the ring-opened form merocyanine (**MC**) were found to be the hydrogen bonding and polarity, aggregation effect, and viscosity, respectively. A newly modified Jablonski diagram was proposed to clarify the emission behaviors of spiropyran influenced by solvent polarity and isomerization. Meanwhile, the solvent effect could also be extended to a solid polymer matrix (six kinds of polyethylene glycol (PEG) with different molecular weights), which is proposed to be defined as the generalized solvent effect. Accordingly, we have demonstrated that the unique fluorescence properties of spiropyran are dominated by the generalized solvent effect. The security information storage capacity of the simulated quick response (QR) code sensor combined with **SP** for anti-counterfeiting was significantly improved to six dimensions in taking advantage of the former theoretical analysis.

## 1. Introduction

Spiropyran (**SP**) is one of the earliest and most widely studied organic photochromic materials, and its photochromic behavior is induced by reversible molecular isomerization [1]. Meanwhile, due to isomerization, **SP** exhibits unique fluorescence characteristics, which can be switched between non-fluorescent ring-closed form SP and red fluorescent ring-opened form merocyanine (**MC**) [2]. The tunability and photoswitch properties of **SP** have attracted much attention from researchers and have been widely used in such fields as super-resolution imaging [3], bioimaging [4,5], chemical sensing [6], ion detection [7], optical recording [8], and especially in the optical anti-counterfeit related to the quick response (QR) code. The QR code, which is a two-dimensional identification code with high and tunable data storage capacity (25–4296 characters), has been used as commercial anti-counterfeiting code sensing and increasingly cannot be repeatably authenticated online based upon the counted verification times [9]. But one can still mix the spurious with the genuine through the security hole of counterfeiting unused QR security codes. The ring-opened form of **SP** typically exhibits an extremely high molar absorption coefficient, making it suitable for a wide range of stimuli-responsive color modulation. Additionally, its color-changing speed is remarkably fast, and it is one of the rare dual-functional molecules capable of both color change and luminescence. These characteristics make **SP** highly suitable for applications in external displays and scenarios requiring instantaneous information modulation. Furthermore, the synthesis and modification of **SP** derivatives involve a certain level of research complexity, which can, to some extent, reduce the risk of imitation or counterfeiting. Combining QR code with **SP** can significantly improve the replication difficulty and its anti-fake ability by fabricating fibers or inks with color or emission change properties [10,11]. Unlike the excellent photochromic properties of **SP** derivatives with high molar absorptivity, their fluorescence quantum yields are generally low. Moreover, almost all small molecule **SP** derivatives will suffer from the aggregation-caused quenching (ACQ) effect, which limits the further application of **SP**. Therefore, various attempts have been carried out to enhance its fluorescence. For example, the formation of nanoparticles [12,13,14,15,16] with **SP** in the core will weaken the ACQ effect of **SP** by isolating it from solvents and demonstrate excellent dual-color fluorescence, and the emission of the non-fluorescent **SP**, as mentioned, will be improved significantly, which is comparable to that of **MC**. Metal ions [17,18], some special anions [19,20,21,22], and amino acids [23,24] can also promote the emission of **MC** through complexation, which is beneficial to the thermodynamic equilibrium of **SP** → **MC**. Micellization [25,26] is another effective means to achieve the fluorescence enhancement of **MC** by providing a local spatial hindrance. Although there are many valid candidate methods to improve the emission of **MC** and a dual photochemical process (photochromism and photoluminescence) model of **SP** has been proposed based upon the modified Jablonski diagram [3,27,28], few researchers draw attention to the essential reason that affects the emission. In our previous study, a mechanism of three factors (i.e., polar environment, solvent-free, and steric hindrance effect) was proposed by using **SP**-modified filter paper [29]. Actually, solvents, as well as the solid matrices in which intermolecular interactions may play an important role, are indispensable for the applications of most **SP** derivatives. The solvent effect is, therefore, not negligible. Meanwhile, in some subsequent studies, **SP** in non-polar matrices can also display strong luminescence by increasing the concentration of **MC** through long-term irradiation [6]. In other words, the necessity of a polar environment is doubtful. 

Based upon the above controversy, a structurally very simple **SP** derivative containing an ethyl group in its molecule was employed for the evaluation of its fluorescence properties in this study. The solvent effect on the fluorescence of **SP** was investigated and evaluated in liquid solvents and extended to a solid polymer matrix systematically by simply introducing Hansen solubility parameters, viscosity, and hydroxyl value (OHV). With the aid of molecular orbital theory and the Jablonski diagram, the spectral shift associated with the energy level changes and energy conversion was clarified. Hydrogen bonding and polarity were considered the main causes for a blue shift of the **MC** emission, while its fluorescence efficiency was mainly determined by solvent or matrix viscosity. In addition, the aggregation effect of **SP** itself could cause a red shift and luminescence efficiency decrease in the **MC** emission. Subsequently, a 2 × 2 square grid with two polymer components of different polarities was prepared according to the generalized solvent effect, as proposed, and the corresponding simulated QR code dramatically demonstrated a three-state transformation with additional four dimensions of foreground and background color changes (two in color, one in emission, and the other one in contrast). The simulated QR code exhibited six dimensions of security information storage with enhanced anti-counterfeiting sensing ability. The evaluation system in this paper was highly compatible with **SP** derivatives and also suitable for other fluorescent sensing materials. It can significantly improve the anti-counterfeiting sensing ability of the traditional QR code sensors. It may help to end long-standing disputes over the reasons for generating the unique fluorescence characteristics of **SP** and can also provide a solid theoretical basis and reliable parameters for further development and applications of the fluorescence properties of **SP**.

## 2. Results and Discussion

### 2.1. The Unique Fluorescence Properties of SP

**Scheme 1:**
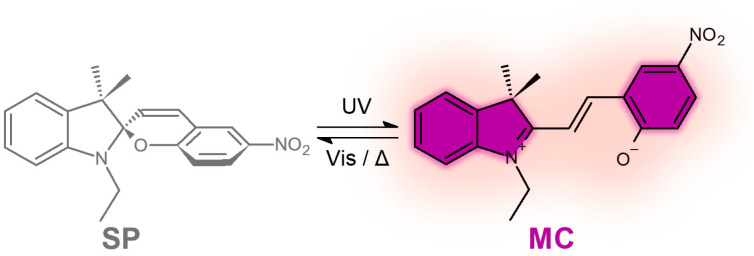
Photoisomerization process between the two isomers of **SP**. The synthesis and characterization of **SP** are shown in Scheme S1, Figures S1 and S2.

**Figure 1:**
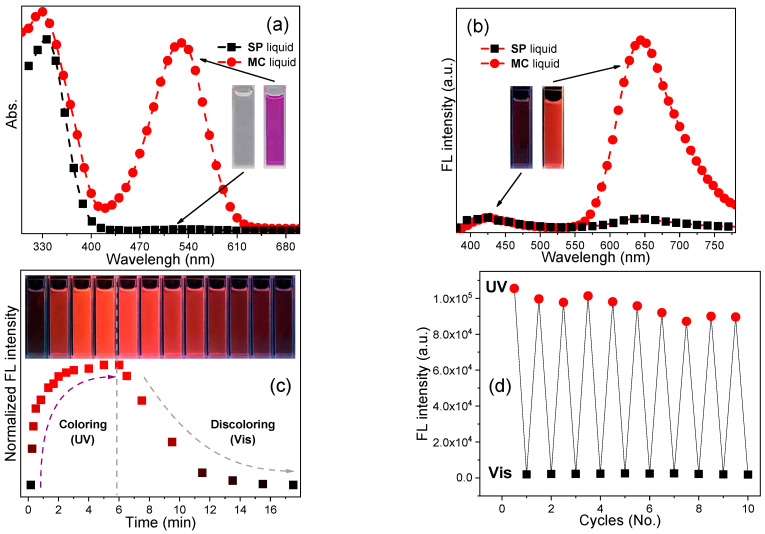
The spectral properties of **SP** (7.5 × 10^−5^ mol/L) in methanol: (**a**) absorption spectra (10 min Vis irradiation for **SP**, and 10 min UV irradiation for **MC**, the inserts were actual photographs of the experimental samples); (**b**) emission spectra (ibid); (**c**) coloration and discoloration processes (the inserts, from left to right, display the actual experimental sample photographs of the coloring process at 0, 2, 4, and 6 min, and the discoloring process at 0.5, 1, 2, 4, 6, 8, 10, and 12 min, respectively); (**d**) recyclability, the sample was repeatedly treated with 10 min Vis or UV.

**Figure 2:**
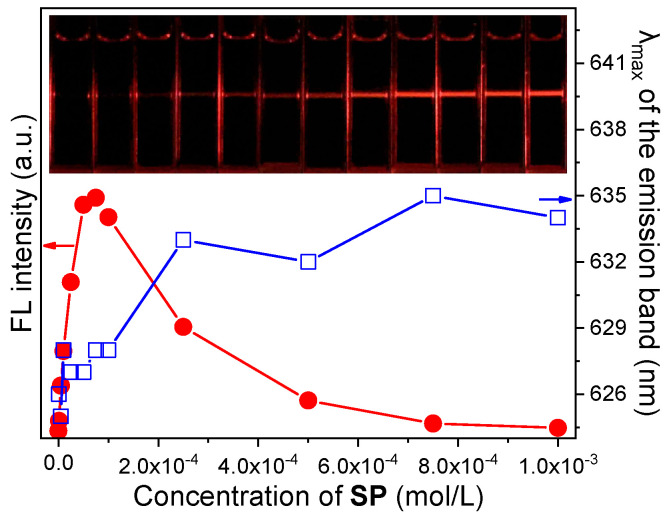
Changes in the emission peak intensity and λ_max_ along with the **SP** concentrations in methanol after 10 min irradiation using a 365 nm UV lamp. The inserted photographs refer to their corresponding Tyndall effect. The original spectral data can be found in Figure S3.

The unique fluorescent properties of **SP** compounds are mainly derived from their isomerization ability (Scheme 1). There are many factors that can affect the isomerization equilibrium of **SP** ⇌ **MC**, such as light [30], solvents [31], temperature [32], force [33], etc. Especially the stimulation of light, with ultraviolet (UV, e.g., 365 nm) irradiation, the colorless (peak wavelength, λ_max_ = 335 nm) and non-fluorescent (λ_max_ = 457 nm, very weak) **SP** will be forced into the colored (λ_max_ = 560 nm, varying with the solvent type changes) **MC** with red light emission (λ_max_ = 646 nm) in methanol, and the process could be reversed when switching to visible light (Vis) irradiation (Figure 1a,b). It could be clearly observed that the wavelength difference between the λ_max_ of the emission of **SP** and **MC** was about 189 nm, and the intensity difference could reach several orders of magnitude. Meanwhile, the fluorescence switching process between **SP** and **MC** under UV or Vis irradiation is rapid and continuous. It could be completely proceeded within 10 min (Figure 1c) and maintain high fluorescence emission even after 10 cycles (Figure 1d). The fluorescence intensity of **MC** would increase linearly at the beginning and then gradually decrease with the solution concentration increasing and the Tyndall effect growing, while λ_max_ showed a continuous red shift. Actually, there exists a maximum value of the fluorescence intensity at the solution concentration of 7.5 × 10^−5^ mol/L, which shows the ACQ effect and indicates that **MC** aggregates significantly and suffers from concentration self-quenching at high concentration (Figure 2). It is noteworthy that a weak emission appears at 646 nm in the fluorescence spectrum of **SP** (Figure 1b), which is attributed to the forming of **MC** by using high-energy UV (365 nm) excitation light of the fluorospectrophotometer. According to the literature [3], such interference from photoisomerization could be reduced by choosing an excitation light located at the absorption valley of **MC** (about 433 nm), while it all depends on the experimental condition and methods.

### 2.2. The Solvent Effect on the Fluorescence Emission Spectra and UV–Vis Absorption Spectra of SP

**Figure 3:**
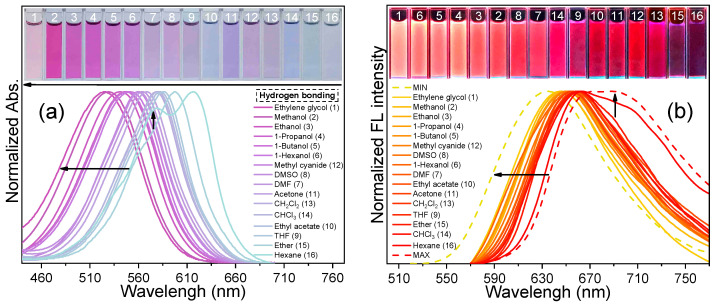
The normalized absorption (**a**) and fluorescence emission spectra (**b**) of **SP** (7.5 × 10^−5^ mol/L) and their corresponding photographs in 16 solvents after 10 min UV irradiation. The same number refers to the same solvent. MIN and MAX in (**b**) refer to the spectra of glycerol and hexane with the minimum and maximum λ_max_ values of all solvents, respectively. The original spectral data of (**a**,**b**) can be found in Figure S4a and S4b, respectively.

Typically, the emission and UV–Vis absorption spectra were obtained from a polar solvent (e.g., methanol). As mentioned above, solvent polarity dramatically affects the spectral behaviors of organic fluorescent compounds, including **SP**. For instance, Abdollahi et al. [34] investigated the solvatochromic behavior of spiropyran in both aprotic and protic solvents, revealing a pronounced dependence of the solvatochromic response on hydrogen bonding and solvent polarity in protic environments. In contrast, the dispersion solubility parameter and solvent polarity emerged as the dominant factors governing the behavior in aprotic solvents. Lin et al. [35] reported the solvent polarity effects on the mechanochemistry of spiropyran ring opening, highlighting the importance of the solvent environment in mechanochemical reactions. Thus, 16 kinds of solvents with different polarities were applied to investigate their influence on **MC**. In terms of the absorption spectra (Figure 3a), a significant blue shift (about 100 nm) of the absorption peak position and a gradually increasing tendency of the absorbance are observed along with an increase in the solvent polarity and hydrogen bonding force (according to the polar and hydrogen bonding components of Hansen solubility parameter). Note that the absorbances of **MC** in solvents after ethanol (e.g., methanol, ethylene glycol) decrease significantly, which might be induced by the exorbitant hydrogen-bonding force of these solvents. Similar phenomena occurring in water had already been reported in our previous study, but water had not been considered for investigation in this study due to the poor solubility of **SP** [36,37]. As for the fluorescence emission spectra (Figure 3b), the red emissions with large Stokes shift affected by the solvent also exhibit a blue shift as far as 56 nm, similar to their corresponding UV–Vis absorption spectra. However, the blue shift of the emission spectra in solvents is slightly different from the increasing trend of their corresponding hydrogen bonding force, which inspires us to investigate the interaction between the solvents and **MC** to discover the reasons that cause deviation. It is worth emphasizing that, in comparison to our previous research [36], which primarily focused on the influence of solvents on the photochromism and solvatochromism of **SP** in solutions, the present work extends the investigation to photoluminescence (fluorescence emission) and solid polymer matrices. Additionally, we propose a generalized solvent effect model and demonstrate its application in dynamic QR code anti-counterfeiting, an area that has not been explored in previous studies. These findings will be systematically presented and discussed in the following sections.

### 2.3. Application of Molecular Orbital Theory to the UV–Vis Absorption Spectra of MC

**Scheme 2:**
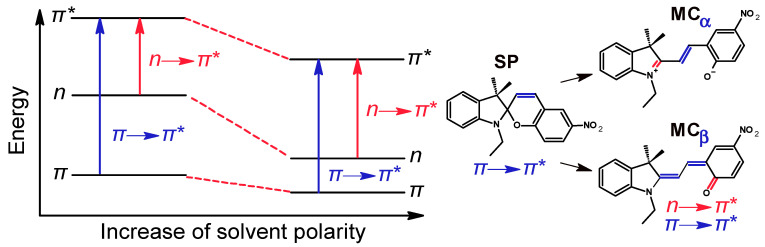
The influence of solvent polarity on the orbital energy of π–π* and n–π* electron transition corresponding to the characteristic molecular structure of **SP**, **MC**_α_, and **MC**_β_.

Since the UV–Vis absorption spectrum and the fluorescence emission spectrum of the same compound are usually mirror-symmetrical, a study on the solvent effect of the former is of great instructional significance for discovering the change rule of the latter in solvents. On the basis of the molecular orbital theory, the UV–Vis absorption of organic compounds is generated by the transition of valence electrons in the molecules. After the photons are absorbed by the molecules, the bonding orbital electrons will jump to the antibonding orbital to form an excited state, and the increased solvent polarity can help reduce the orbital energy. As shown in Scheme 2, for the π-π* transition of electrons in the -C=C- of the ring-closed **SP**, the energy reduction in the π orbital is lower than that in the π* orbital, and the absorption spectra display a red shift; with regard to the n–π* transition of electrons in the -C=N- (**MC**_α_) and -C=O (**MC**_β_) of the ring-opened **MC** (two forms in general, one form is the zwitterionic **MC**_α_; the other form is the quinoid **MC**_β_) [38,39], the energy reduction of the n orbital is higher than that of the π* orbital and the absorption spectra exhibit a blue shift. It should be noted that in aromatic systems, π–π* transitions involve delocalized π electrons across the entire molecular framework rather than being localized to a specific bond. Similarly, n–π* transitions in **MC** involve lone pair electrons from heteroatoms (e.g., N, O) and are also delocalized. In particular, the structural characteristics of spiropyran isomers are highlighted in the schematic diagram, but their delocalization is not discussed or demonstrated. Overall, **SP** mainly exhibits π–π* transition because its structure is an aromatic system, while **MC** may have π–π and n–π transitions at the same time because its structure contains C=N and C=O bonds. In particular, the blue shift of the absorption spectra of **MC** in solvents with different polarity is derived from the changes in the n–π* transition in its structure.

### 2.4. Hansen Solubility Parameter

**Figure 4:**
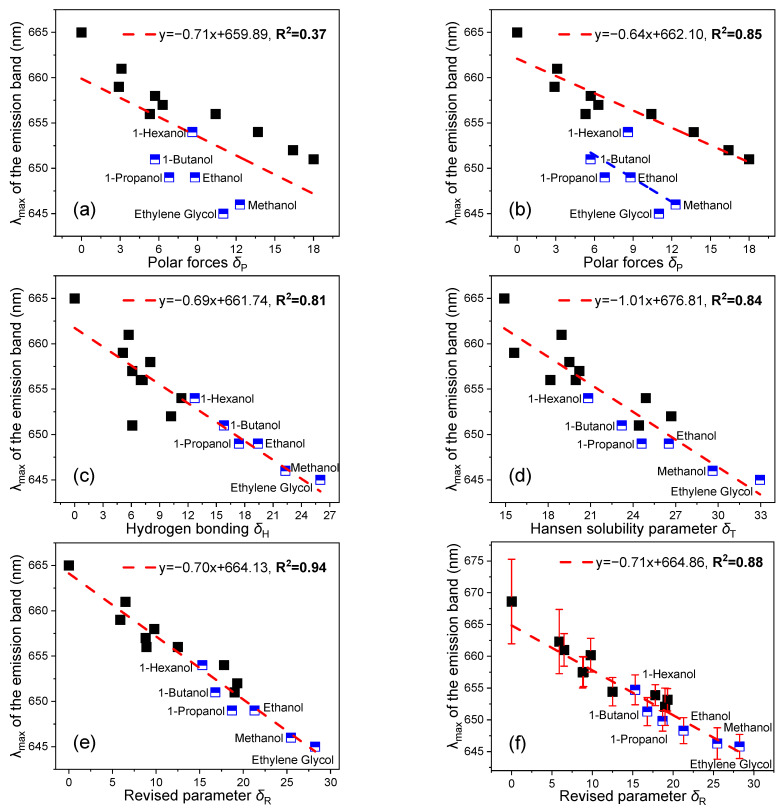
The relationships between λ_max_ of the emission band of **MC** in 16 solvents and different parameters: (**a**,**b**) polar force *δ*_P_; (**c**) hydrogen bonding *δ*_H_; (**d**) Hansen solubility parameter *δ*_T_; (**e**,**f**) a revised parameter *δ*_R_ derived from *δ*_T_, the fittings through *δ*_T_ were obtained in using statistical method (**e**) and standard deviation method (**f**), respectively. The original spectral data used for analyses and fittings can be found in Figure S5. Parameters and λ_max_ values in different solvents used above and in Figure S5 are listed in Table S1.

Based upon the above discussion, the solvent polarity might also have a significant impact on the peak positions of the emission spectra of **MC**. It is known that the solvent effect on fluorescence emission is caused by the intermolecular interaction of solvent and solute, which is mainly related to the properties of the solvent itself (refractive index, dielectric constant, polarity, etc.) and is also induced by their special chemical interaction (hydrogen bonding, complexation, etc.). The impact extent of different solvents is usually illustrated by the Lippert–Mataga equation [40]. However, the calculation process through the Lippert–Mataga equation is too complicated, for it contains many parameters and variables. Accordingly, a simple and reliable parameter that can characterize the intermolecular interaction and replace this equation is urgently needed to measure the solvent effect on the emission of **MC**. Solubility parameters were considered to be excellent candidates. Among various solubility parameters (e.g., Hildebrand’s one-component solubility parameter [41], Bagley’s two-component solubility parameter [42], Hansen’s three-component solubility parameter [43]), Hansen’s three-component solubility parameter *δ*_T_ (*δ*_T_^2^ = *δ*_D_^2^ + *δ*_H_^2^ + *δ*_P_^2^), including dispersion component *δ*_D_, hydrogen bonding component *δ*_H_, polar component *δ*_P_ dramatically shows good effectiveness and practicability. Hence, the Hansen solubility parameter and all its components were employed to analyze the wavelength changes and pick out the main factor. *δ*_P_ related to the solvent polarity was the first to be taken into consideration. As expected, the λ_max_ has a blue shift trend with the increase in *δ*_P_, while the linearity of the fitting is poor (Figure 4a). However, the λ_max_ of alcohol solvents tends to change independently, and the goodness of fit (also known as the coefficient of determination) could be improved by excluding these solvents (Figure 4b). It reveals that the blue shift of the emission spectra of **MC**, similar to that of the UV–Vis absorption spectra, is also caused by the changes in the n–π* transition in its structure. On the one hand, alcohol solvents with hydroxyl groups have high hydrogen-bonding values; on the other hand, **MC** is a very sensitive hydrogen-bonding receptor. Therefore, *δ*_H_ was reselected for linear fitting, and the linearity significantly improved compared with *δ*_P_. Also, the λ_max_ values of alcohol solvents return to the fitting line (Figure 4c). Indeed, the fitting results illustrate that the use of Hansen solubility parameter components is of good guiding significance. With the trial of *δ*_T_, the goodness of fit is increased to 0.84 (Figure 4d). But the improvement is very limited, which may be due to the fact that dispersion force is induced by the instantaneous dipole in any molecule, and no significant difference exists in *δ*_D_ between different solvents.

Briefly, the most important factor that affects the λ_max_ of **MC** in solvents is hydrogen bonding, and the secondary factor is solvent polarity, while dispersion force does not contribute much to the blue shift of the emission spectra. On this account, *δ*_D_ can be removed from *δ*_T_, and a revised parameter *δ*_R_ (*δ*_R_^2^ = *δ*_H_^2^ + *δ*_P_^2^) is obtained. The experimental data are then fitted by *δ*_R_, and the R^2^ has been greatly improved as predicted (Figure 4e). Beyond that, an empirical polarity parameter E_T_(30) has also been tried to take the place of *δ*_T_. Although the goodness of fit of E_T_(30) is better than that of *δ*_T_, it is not comparable with *δ*_R_ (Figure S6a).

Since sufficient samples were collected for analysis, the above fitting is based on the statistical methods, and the emission peak wavelength value with the highest frequency of all is set as the λ_max_. If the standard deviation method is employed, the R^2^ will slightly drop when using *δ*_R_. It appears that the errors gradually increase with *δ*_R_ or solvent polarity declining (Figure 4f and Figure S6b). This should be associated with the aggregation effect of **MC**. When the solvent is non-polar, such as hexane, **MC** strongly aggregates and forms head-to-head J-aggregates (amphoteric structure) [44,45]. The absorption and emission spectra of **MC** will show an obvious red shift similar to the phenomenon observed in Figure 2. The aggregation effect is so strong that it can be observed even in polymer matrices [46].

### 2.5. Isomerization Process Related to Energy Level Transition and Simplified Jablonski Diagram

Except for λ_max_, another key issue is the fluorescence quantum yield. According to the Jablonski diagram [47,48], the fluorescence of most organic luminophores is derived from the radiative transition. Reducing energy loss by blocking non-radiative transition will help to improve the efficiency of the fluorescence process. Typically, there are three main pathways in which **MC** can dissipate energy through the non-radiative relaxation processes: a. vibrational relaxation; b. internal conversion; c. non-radiative de-excitation (including external conversion and solvent-induced isomerization reaction). Regardless of the energy consumed by the radiative luminescence, most of the stored excitation energy will be dissipated non-radiatively by the release of thermal energy through pathway c. The aforementioned λ_max_ is mainly affected by vibrational relaxation and internal conversion, while the external conversion and isomerization reaction are the most direct factors affecting the fluorescence intensity and fluorescence quantum yield. According to the thermodynamic principle proposed in the literature [36], when strong polar **MC** interacts with solvent molecules, its energy level will gradually decrease as the polarity of the solvent increases. Usually, the thermodynamic energy level of **MC** is lower than that of **SP** in polar solvents and higher in non-polar solvents. Meanwhile, the energy level difference between the two isomers varies with solvent polarity. Although the classical Jablonski diagram effectively describes the photophysical processes of materials, it falls short of adequately explaining the complex photophysical and photochemical processes of organic molecules involving photochemical reactions. Given that the spiropyran molecules discussed in this study do not involve phosphorescence and that spiropyran, as a photoisomerization system, is strongly influenced by solvent effects, we have further simplified and modified the Jablonski diagram. Specifically, we omitted the phosphorescence process, reduced the number of energy levels, and integrated the photoisomerization process along with the effects of solvent interactions into a single diagram. This modification aims to clearly and concisely elucidate the photophysical and photochemical processes of spiropyran molecules under the influence of solvent effects.

**Scheme 3:**
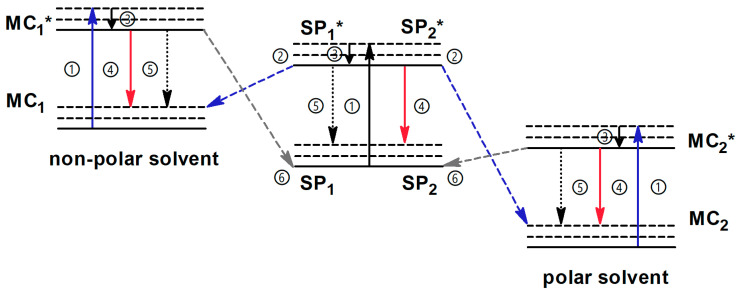
A simplified Jablonski diagram with consideration of the solvent polarity and isomerization of **SP**. The **SP**_1_*/**SP**_2_* and **MC**_1_*/**MC**_2_* refer to the excited state of the round states **SP**_1_/**SP**_2_ and **MC**_1_/**MC**_2_ stimulated by UV, respectively. Different solid and dashed arrows denote different processes: (1) absorption (e.g., 365 nm UV); (2) photoisomerization, SP* → **MC**; (3) vibrational relaxation; (4) fluorescence (radiative transition); (5) external conversion (non-radiative de-excitation); (6) solvent-induced isomerization (non-radiative de-excitation), MC* → **SP**.

As shown in Scheme 3, if **SP** is irradiated with 365nm UV, the ground state **SP**_1_/**SP**_2_ will be excited to the excited state **SP**_1_*/**SP**_2_* (process 1). Since the energy levels of the ground state **MC**_1_/**MC**_2_ are all lower than that of **SP**_1_*/**SP**_2_*, **SP**_1_*/**SP**_2_* → **MC**_1_/**MC**_2_ (photoisomerization, process 2) is a thermodynamic spontaneous process. Subsequently, **MC**_1_/**MC**_2_ is also excited to the excited state **MC**_1_*/**MC**_2_* by UV (process 1), and **MC**_1_*/**MC**_2_* will fall back to the lowest vibrational level of the first electronic excited state rapidly after vibrational relaxation (process 3). Then, part of the rest of the energy is converted into radiative transition and emitted as fluorescence (process 4), and the other energy is dissipated non-radiatively by external conversion (inelastic collision between **MC** and solvent or other **MC** molecules, process 5) and solvent-induced isomerization reaction (process 6) until the process is balanced. The energy level difference (energy barrier) of **MC**_1_* → **SP**_1_ in non-polar solvent is higher than that of **MC**_2_* → **SP**_2_ in polar solvent, so is the reaction rate and energy consumption. The solvent effect on the reaction rate of MC* → **SP** (charge density of the reaction decreases) can also be judged by Hughes–Ingold rules [49], and the reaction rate tends to decrease with the increase in solvent polarity. All these factors will lead to a decrease in the fluorescence quantum yield of **MC** in non-polar solvents. Meanwhile, with consideration of the ACQ effect of **MC** related to the concentration, the concentration of all samples is controlled below the self-quenching concentration, and a new parameter *K* = *F*/*A* based on the fluorescence emission peak intensity (*F*, located at ~650 nm) and absorbance (*A*, located at ~550 nm) of **MC** is proposed (see Figure S7 and Table S2 in the Electronic Supplementary Information for the selection and demonstration process of *K* values). The change in *K* values is concentration-independent but dependent on the fluorescence quantum yield. Note that here, it is not recommended to use the integrated fluorescence intensity and the absorbance of the excitation light at 365 nm to calculate the fluorescence quantum yield for correlation analysis in view of the following issues: a. the excitation light can cause a strong isomerization reaction; b. the absorbance of the excitation light at 365 nm is induced by both of the **SP** and **MC**; c. when the solvent is non-polar, the fluorescence emission spectrum of **MC** will shift to the near-infrared region, which is beyond the measurement and calibration range of most fluorescence spectrophotometers. So, it is extremely hard to calculate the integrated fluorescence intensity accurately.

### 2.6. Viscosity

**Figure 5:**
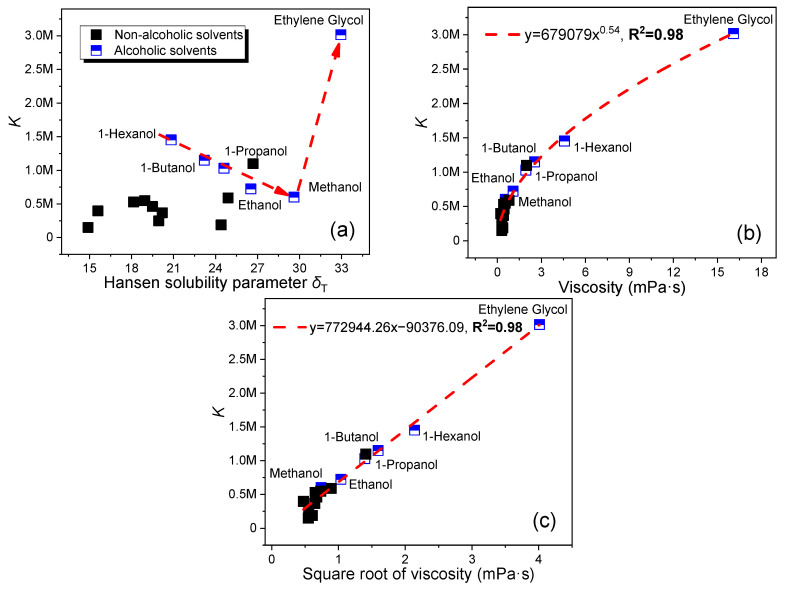
The relationships between *K* of **MC** in 16 solvents and different parameters: (**a**) Hansen solubility parameter *δ*_T_; (**b**) solvent viscosity; (**c**) square root of the solvent viscosity. *A*, *F*, *K*, *δ*_T_, and viscosity values in different solvents used above are listed in Table S2.

It is generally known that the dispersion force is the main force between the solute and the solvent molecules in a non-polar solvent. It also affects the fluorescence intensity and cannot be simply ignored. Since the fluorescence quantum yield of **MC** is relevant to the solvent polarity, *δ*_T_ is employed for fitting with *K* values in different solvents based on the above discussion (Figure 5a). However, only an increasing trend is found between them, and no obvious functional relationship exists. But on closer inspection, it appears that *K* values gradually decrease first and then rise unexpectedly with the increase in *δ*_T_ of the alcohol solvents, which is completely consistent with the viscosity trend of the alcohol solvents. Further fitting between the viscosity and *K* values by power function y = a × x^b^ exhibits an excellent R^2^ as high as 0.98 (Figure 5b). Meanwhile, the power exponent of the fitting function is approximately equal to 0.5, similar to solubility parameters, which are defined as the square root of the cohesive energy density. As expected, the goodness of the linear fit with the trial of the square root of the viscosity turns out to be as excellent as the viscosity. This indicates that the fluorescence quantum yield of **MC** in solvents is mainly determined by the solvent viscosity. It might be due to the fact that viscosity is an internal friction force produced by hindering the relative motion between molecules through intermolecular cohesion. The choice of a solvent with high polarity or viscosity, which helps to reduce the energy barrier and inelastic collision, will facilitate the fluorescence emission of **MC**. Solubility parameters may play a key role as guidance. However, the existing solubility parameters (e.g., *δ*_T_) cannot accurately reflect the solvent effect on the fluorescence efficiency of **MC**. Then, glycerol with extremely high viscosity and polarity was tested, and its experimental *K* value (Table S2) was close to the predicted *K* value through the fitting function in Figure 5c. In addition, the *K* value of glycerol is 100 times that of hexane. It can be predicted that the fluorescence intensity and fluorescence quantum yield of **MC** should also be promoted notably with the increase in the solute viscosity or solid matrices. Actually, plenty of studies have been reported to support this argument [37,50,51,52].

### 2.7. Application of the Solvent Effect in Polymer Matrices

Usually, the solvent effect is proposed based on liquid solvents. In practice, however, **MC** is often used as nanoparticles or in polymer matrices. Investigation of the uncertain fluorescence of **MC** in solid environments is of great significance and can also guide the following QR code application. So, work on extending the concept and application scope of the solvent effect in a narrow sense from liquid solvents to solid solvents (it can also be regarded as extremely viscous liquids) was carried out to explain the luminescence behavior of **MC**. Viscosity is no longer an important parameter at present because the solid viscosity is very high and shows little difference from each other. Also, the ACQ effect of **MC** in a polymer matrix is weakened by the barrier of polymer chains [29]. It can be observed through the comparative high fluorescence intensity in all polyethylene glycol (PEG) samples. More attention should be paid to λ_max_, and the solubility parameter was considered the preferred evaluation method. Since there is no unified standard for testing the solubility parameters of the polymers and their corresponding parameters given in the literature do not have reliable correlation, another important indicator of PEG, OHV [53] (it refers to the number of milligrams of KOH equivalent to the hydroxyl content of 1g sample, usually expressed in units mgKOH/g, the higher the PEG molecular weight, the lower its OHV), is selected.

**Figure 6:**
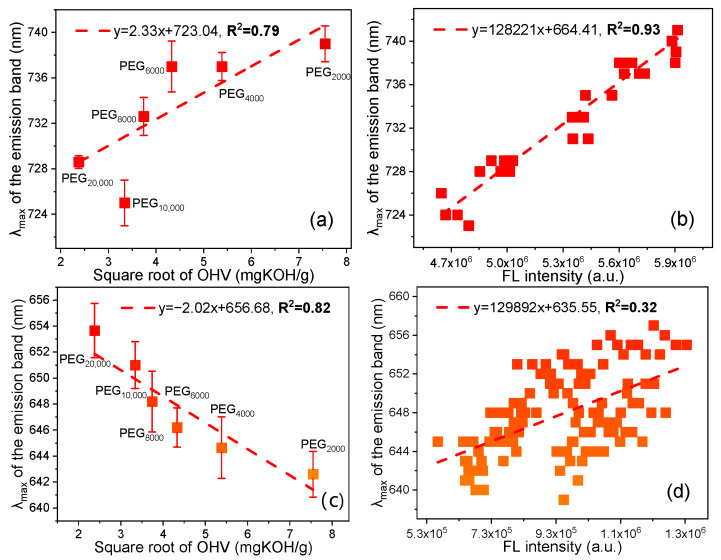
The relationships (**a**,**c**) between λ_max_ of the emission band of **MC** (c_SP_ = 10^−7^ mol/g) in PEG ((**a**) solidified state, (**c**) seven d after curing) with different molecular weights (Mn = 2000, 4000, 6000, 8000, 10,000, 20,000) and square root of OHV. (**b**,**d**) represent the relationship between λ_max_ from (**a**,**c**) and all its corresponding fluorescence intensities, respectively. The original spectral data of (**a**,**b**) and (**c**,**d**) can be found in Figures S8 and S9, respectively. OHV values of PEG with different molecular weights used above are listed in Table S3.

With reference to the solubility parameter, the square root of the OHV, i.e., OHV^0.5^, is applied to the linear fitting. As shown in Figure 6a, when the **SP** contained PEG_2000_ ~ PEG_20000_ are solidified, λ_max_ values show a significant red shift with the increase in OHV^0.5^, far beyond the maximum value measured in hexane. It is known that the higher the OHV, the higher the hydroxyl content, and the stronger the hydrogen bonding. According to the previous analysis, λ_max_ should demonstrate a blue shift, which seems to be contrary to our results. However, all test samples concerning the **SP** containing PEG are analyzed, and a linear relationship between λ_max_ and its corresponding fluorescence intensity is found (Figure 6b), which fully indicates that **MC** will strongly aggregate very soon after the PEG is solidified. For this moment, the dominant factor leading to a red shift of λ_max_ is the aggregation effect of **MC** (as a result of the weakening of ACQ effect of **MC** in solid, the higher the **MC** concentration is, the stronger the fluorescence intensity and the aggregation grow, and the further the emission spectrum shifts to red). So, the PEG samples were placed in the dark for 7 days to stabilize the internal environment and chemical structure. Then, the changing trend of λ_max_ predictably reverses and exhibits a linear blue shift (Figure 6c). The linear relationship between λ_max_ and its corresponding fluorescence intensity no longer exists (Figure 6d), which exactly adheres to our previous inferences. One can claim that the principle of the solvent effect on **MC** in liquid solvent is also applicable to the solid polymer matrix, which can be defined as the generalized solvent effect.

**Figure 7:**
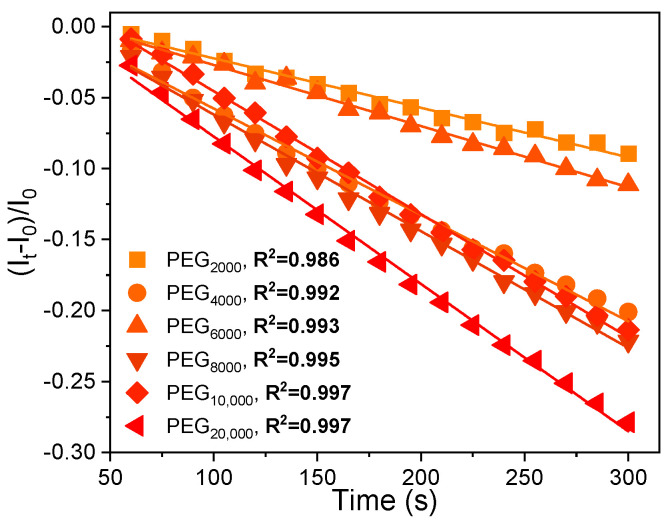
Photobleaching of **MC** (c_SP_ = 10^−7^ mol/g) in PEG with different molecular weights (Mn = 2000, 4000, 6000, 8000, 10,000, 20,000). The curves were obtained from Figure S8a′–f′.

Meanwhile, a special phenomenon has arisen. As the molecular weight of PEG decreased and the OHV increased, the photobleaching of **MC** gradually weakened (Figure 7). Similar phenomena also occur in solvents: the stronger the polarity and hydrogen bonding of solvents, the weaker the photobleaching of **MC**. In alcohol solvents, photobleaching is even negligible. As proved in Figure 4, the fluorescence of **MC** has been strongly influenced by solvent polarity and hydrogen bonding, and hydrogen bonding can stabilize the molecular structure of the excited **MC**. All this illustrates again that the environment of strong polarity and hydrogen bonding could help reduce the photobleaching and is beneficial to the fluorescence emission of **MC**.

### 2.8. QR Anti-Counterfeiting Sensing

Extending the concept and application scope of the solvent effect into the solid polymer matrix has high potential application value, such as data storage, anti-counterfeiting, super-resolution imaging, etc. For instance, Zhi et al. [54] reported a trident anchoring strategy for highly specific and dynamic imaging of lipid droplets, demonstrating the potential of fluorescent probes in biological research. Similarly, Shen et al. [55] developed chiral phosphorescent microflowers from Au nanoclusters for dual-mode pH sensing and information encryption, highlighting the potential of fluorescent materials in anti-counterfeiting applications. In fact, **SP** still shows solvatochromism and photochromism in matrices. This exciting new finding of significant spectral changes in **SP** in a solid polymer matrix motivated us to employ this system to develop a photochromic dye, which could be used for anti-counterfeiting sensing in multiple dimensions. 

**Figure 8:**
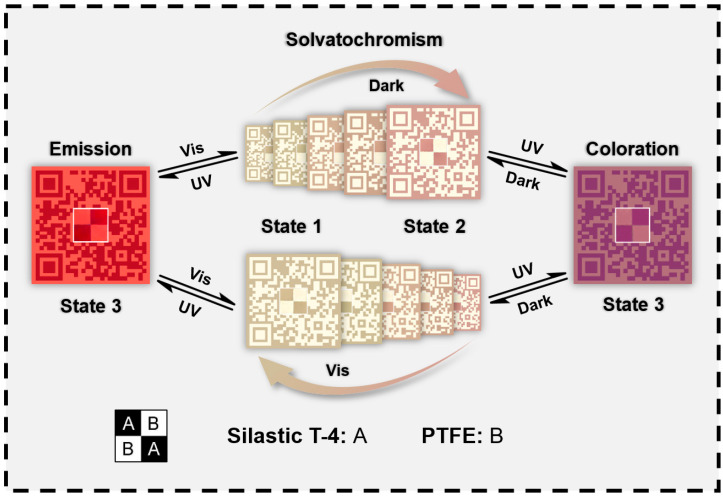
QR code-sensing application. All QR code sensors are simulated based on the color changes in the 2 × 2 polymer square grid under different stimulations. Without UV irradiation (middle), the non-fluorescent 2 × 2 polymer square grid can produce weak reversible color changes with dark background and light foreground in visible light (middle left, state 1) or dark (middle right, state 2). With UV irradiation (left and right, state 3), the state of the square grid could be reversed with presentation of the dark foreground and light background both in its emission (left) and coloration (right). Meanwhile, the process is also reversible. Three states exist, along with one contrast change and four kinds of color changes.

Here, Silastic T-4 (polar, contains hydroxyl, according to Fourier transform infrared spectroscopy (FTIR) in Figure S1b) and polytetrafluoroethylene (PTFE, non-polar) were selected in consideration of their huge polarity differences. Firstly, these two polymers were prepared into a 2 × 2 square grid with a white background, and their corresponding color was simulated as a QR code sensor. Normally, the transparent Silastic T-4 is beige due to the refractive index, while PTFE is opaque milky white (state 1 in Figure 8). Meanwhile, both of them are non-fluorescent. The doping of **SP** into the two polymers has no apparent influence on their color. When the 2 × 2 square grid was placed overnight in the dark, the beige polar part turned light pink, while the non-polar part stayed the same (state 2 in Figure 8). This transformation could be reversed in visible light, and the whole process was demonstrated in the simulated QR code sensors with real 2 × 2 square grid inserts. It is well known that this reversible process is equal to the solvatochromism in solvents, as reported. After 10 min UV irradiation, the color of the entire square grid changed with the massive formation of **MC**. Silastic T-4 and PTFE showed dark pink and dark purple colors with orange, red, and dark red emissions, respectively. Both their color and fluorescence exhibit high contrast, and all their corresponding simulated QR code sensors can be well recognized (state 3 in Figure 8). Also, the contrast of the foreground and background color has been reversed. Even if the contrast change is excluded, the color information of the same simulated QR code sensor can still change three times in contact with three stimuli (i.e., UV, Vis, matrices, or solid polymer solvents). The dimension of security information storage has been greatly improved and increased to as high as six dimensions. For the preparation, no additional fluorescent sensing materials need to be considered, only the suitable polarity of the matrix. Regarding the recognition, only a miniature UV lamp is needed. The process is also extremely fast. It occurs in a few seconds and shows good recognition within minutes. It can be applied to the color-changing printing ink system in view of the existing efforts for anti-counterfeiting sensing as well. Actually, the application does not stop there; it can still be used in chemical sensing, VOC sensing, environmental monitoring, etc., combined with protonation, Förster resonance energy transfer (FRET), excited-state intramolecular proton transfer (ESIPT), and aggregation-induced emission (AIE) functional groups, etc. As discussed above, the fluorescence properties of **SP** can be extended to more fields. What had been conducted is only a beginning.

## 3. Materials and Methods

### 3.1. Materials and Instruments

All the reagents (Aladdin) and solvents (Sinopharm Chemical Reagent Co., Ltd., Shanghai, China) were purchased commercially and used without further purification. The proton nuclear magnetic resonance (^1^H NMR) spectrum was recorded with a Bruker Advance III 600 spectrometer (Bruker, Karlsruhe, Germany). The FTIR spectra were taken from a Bruker Tensor 27 spectrometer (Bruker, Germany). The UV–Vis absorption spectra were recorded with a UV-2550 spectrophotometer (Shimadzu, Kyoto, Japan). The fluorescence spectra were obtained using a FluoroMax-4 spectrometer (HORIBA Jobin Yvon, Kyoto, Japan, S/N > 16000:1). The fluorescence photographs were captured upon 365 nm UV irradiation. Note that “spiropyran” in this paper can be used to describe the photochromic material spiropyran and its ring-closed form, which is abbreviated as **SP**, while merocyanine can only be used to describe the ring-opened form of spiropyran, abbreviated as **MC**. This expression is common in various references.

### 3.2. Fluorescence Measurements

All the fluorescence spectra were obtained using corrected signals. Subtracting blanks, removing dark noise, and correcting for inhomogeneities in the instrument or detector response give more accurate spectra. If *S* is defined as the signal, correction follows the equation:

*S*_corrected_ = (*S*_measured_ − *S*_dark_ − *S*_blank_) × Correction-factor file

For the emission spectra, an excitation light of 365 nm was employed. And for the excitation spectra, the λ_max_ values of their corresponding emission spectra were used as the emission lights. The slits of the excitation and emission light of the fluorescence spectra were 10 nm and 5 nm, respectively. The integration time is 0.01 s. In order to eliminate the errors induced by artificial operation and solvatochromism, an accumulation method through the stacked scans (20 scans for Figure 4, Figure 5, and Figure 6c, and 5 scans for Figure 6a and Figure 7) was introduced into the measurement of the emission spectra. The isomerization process induced by UV irradiation could be accurately simulated by cyclic fluorescence measurements since the excitation light was always on during the tests. Accordingly, one cycle took about 15 s under current conditions.

### 3.3. Synthesis of Compound SP

The **SP** (1′-ethyl-3′,3′-dimethyl-6-nitrospiro[chromene-2,2′-indoline]) was synthesized using the procedure shown in Scheme S1 according to the literature [56]. A mixture of compound **1** (2,3,3-trimethyl-3H-indole, 3.18 g, 20 mmol) and ethyl iodide (3.12 g, 20 mmol) in ethanol (25 mL) was refluxed under nitrogen at 85 °C for 4 h to obtain the residue after ethanol was removed under reduced pressure distillation. Then, the residue was recrystallized and washed three times with ethanol (15 mL), and a light purple solid of compound **2** (1-ethyl-2,3,3′-trimethyl-3H-indolium iodide, 4.98 g, 79%) was obtained. After that, a solution of compound **2** (4.98 g, 16 mmol), 2-hydroxy-5-nitrobenzaldehyde (2.71 g, 16 mmol), and piperidine (1.38 g, 16 mmol) in ethanol (50 mL) was refluxed under nitrogen at 85 °C for 4 h. The reaction solution was left standing overnight. After purification via recrystallization in ethanol and filtration, a light green powder of the product **SP** (2.12 g, 39.5%) was finally procured. The **SP** was characterized by FTIR (Figure S1) and ^1^H NMR (Figure S2) spectra. FTIR (KBr, cm^−1^): Ar-H, 3062; -CH_3_, 2968, 2906, 1458, 1379; -CH_2_-, 2935, 2867; -C=C-, 1651; Ar-C, 1610, 1576, 1484; -C-O-C-, 1028. ^1^H NMR (600 MHz, CDCl_3_, δ, ppm): 1.16 (m, 6H, -CH_3_×2); 1.28 (s, 3H, -CH_3_); 3.15–3.37 (m, 2H, N-CH_2_); 5.82 (s, 1H); 5.84 (s, 1H, -CH=CH-); 6.56–7.19 (m, 4H, Ar-H in indoline moiety); 7.97–8.05 (m, 2H, Ar-H in chromene moiety).

### 3.4. Preparation of 2 × 2 Polymer Square Grid

The 2 × 2 square grid composed of PTFE and Silastic T-4 polymer containing **SP** was prepared through the following procedure:

- A 2 × 2 square grid pattern with individual square cavities (each measuring 5 × 5 × 3 mm) was fabricated on a PMMA plate using a laser engraving machine. The walls between the cavities were approximately 0.5 mm thick;
- Two precursor solutions, each with an **SP** concentration (c_SP_) of 10^− 7^ mol/g, were prepared using Dow Corning Silastic T-4 transparent silicone and DuPont PTFE emulsion (60% solid content), respectively. These solutions were then poured into the diagonal cavities of the prepared grid, as illustrated in Figure 8. Subsequently, the grid was fully cured at 60 °C for 24 h to obtain the final polymer square grid.

### 3.5. Fabrication and Demonstration of the QR Codes

The QR codes displayed in Figure 8 were created and generated using the QR Code Generation Tool from liantu.com, with a resolution of 300 × 300. The central 2 × 2 square grid within the QR codes, serving as an embedded logo, is an actual photograph (captured under Vis or UV light) of the grid fabricated in Section 2.4. The foreground and background colors of the QR codes were sampled using an eyedropper tool from the PTFE and Silastic T-4 regions, respectively, within the central 2 × 2 square grid of the QR code. For detailed operations and demonstrations, please refer to the discussion in Section 2.8.

### 3.6. Preparation of SP-Contained PEGs

**SP** and PEG with different molecular weights (Mn = 2000, 4000, 6000, 8000, 10,000, 20,000) were co-dissolved in an appropriate amount of dichloromethane and thoroughly mixed. The mixture was then dried and solidified in an oven at 80 °C to obtain **SP**-contained PEGs (c_SP_ = 10^−7^ mol/g) with different molecular weights (Mn = 2000, 4000, 6000, 8000, 10,000, 20,000). The preparation of **SP**-contained PEGs requires approximately 1 h in total, including solvent evaporation, complete curing, and cooling to room temperature.

## 4. Conclusions

The solvent effect was investigated both in liquid solvents and solid PEG matrices. Determinants for red shift and blue shift of the emission spectra of **MC** were linked to the aggregation effect or the hydrogen bonding and polarity, respectively, while viscosity is the most important factor affecting the luminescence efficiency of **MC**. The selected parameters (Hansen solubility parameters, viscosity, and hydroxyl value) have significant advantages in spectral analysis over the Lippert–Mataga equation. The emission behaviors of spiropyran could be well clarified by the newly modified Jablonski diagram. Meanwhile, the solvent effect can not only be regarded as a mechanism of action but also be used to enhance the optical properties of materials. Currently, the generalized solvent effect, as proposed, can effectively improve the anti-counterfeiting sensing ability of the simulated QR codes. To conclude, this generalized solvent effect model and evaluation system exhibit broad applicability, offering a resolution to long-standing debates on the fluorescence mechanisms of **SP**. It not only provides a solid theoretical basis and reliable parameters for the further development and application of fluorescence properties in **SP** derivatives and other fluorescent sensing materials but also proposes a scalable strategy for designing advanced anti-counterfeiting sensors and functional materials/coatings. 

## Data Availability

The original contributions presented in this study are included in the article/Supplementary Material. Further inquiries can be directed to the corresponding author.

## Supplementary Materials

The supporting information can be downloaded at https://www.mdpi.com/article/10.3390/ijms26041531/s1. References [43,57,58,59] are cited in the supplementary materials.
