# Supplementary material for: Generalized Solvent Effect on the Fluorescence Performance of Spiropyran for Advanced Quick Response Code Dynamic Anti-Counterfeiting Sensing
Source: Int J Mol Sci. 2025 Feb 12;26(4):1531. doi: 10.3390/ijms26041531 (PMC11855843; doi:10.3390/ijms26041531)
Supplement: Supplementary file 1 [file ijms-26-01531-s001.zip › ijms-3395823-supplementary.docx]

**Electronic Supplementary Information**

**Generalized solvent effect on the fluorescence performance of spiropyran for advanced quick response code dynamic anti-counterfeiting sensing**

Junji Xuan ^a, b*^, Lingjie Chen ^a^, Jintao Tian^b^

^a^ National Key Laboratory of Marine Corrosion and Protection, Luoyang Ship Material Research Institute, Luoyang 471023, P. R. China.

^b^ School of Materials Science and Engineering, Ocean University of China, Qingdao 266100, P. R. China.

* Correspondence: xuanjunji@gmail.com (J.X.)

**Contents**

1. Synthesis process of **SP**
2. The FTIR spectra of **SP** and Silastic T-4
3. The ^1^H NMR spectrum of **SP**
4. Original emission spectra for Figure 2
5. Original UV-Vis absorption and fluorescence emission spectra for Figure 3
6. Original fluorescence emission behaviors of **SP** in different solvents with time-dependent irradiation for the analysis in Figure 4
7. The relationships between λ_max_ of the emission band of **MC** in 16 solvents and different parameters
8. Values of different solvent parameters and λ_max_ in different solvents
9. The determination of *K*, relevant data and parameters for analysis in different solvents
10. Fluorescence emission behaviors of **SP** contained PEG with different molecular weight curing for 1h
11. Fluorescence emission behaviors of **SP** contained PEG with different molecular weight curing for 7d
12. OHV values of PEG with different molecular weights
13. **Synthesis process of SP**

**Scheme S1.** Synthesis process of **SP**

1. **The FTIR spectra of SP and Silastic T-4**

**Figure S1.** The FTIR spectra of **SP** (a) and Silastic T-4 (b).

1. **The ^1^H NMR spectrum of SP**

**Figure S2.** The ^1^H NMR spectrum of **SP**.

1. **Original emission spectra for Figure 2**

**Figure S3.** Fluorescence emission spectra of **SP** with different concentrations in methanol after 10 min irradiation by using a 365 nm UV lamp.

1. **Original UV-Vis absorption and fluorescence emission spectra for Figure 3**

**Figure S4.** UV-Vis absorption (a) and fluorescence emission spectra (b) of **MC** in different solvents for the normalization of Figure 3.

1. **Original fluorescence emission behaviors of MC in different solvents with time-dependent irradiatio****n for the analysis in Figure 4**

**Figure S5.** (a-p) Fluorescence emission spectra of **MC** in different solvents; (a′-p′) irradiation time-dependent λ_max_ and their corresponding fluorescence intensities of **MC** in different solvents.

1. **The relationships between λ_max_ of the emission band of MC in 16 solvents and different parameters**

**Figure S6.** The relationships between λ_max_ of **MC** in 16 solvents and different parameters: (a) E_T_(30); (b) revised parameter *δ*_R_, where maximum of λ_max_ and minimum of λ_max_ refer to the maximum and minimum values of the emission peak wavelength of **MC** measured in the same solvent, respectively.

1. **Values of different solvent parameters and λ_max_ in different solvents**

**Table S1** Values of different solvent parameters and λ_max_ in different solvents

| Solvents | λ_max_  (nm) | Et(30) | Hansen solubility parameter | | | | Revised parameter  *δ*_R_ |
| --- | --- | --- | --- | --- | --- | --- | --- |
|  |  |  | *δ*_D_ | *δ*_P_ | *δ*_H_ | *δ*_T_ |  |
| Glycerol | 631 | 57.0 | 17.4 | 12.1 | 29.3 | 36.2 | 31.7 |
| Ethylene Glycol | 645 | 56.3 | 17.0 | 11.0 | 26.0 | 33.0 | 28.2 |
| Methanol | 646 | 55.4 | 15.1 | 12.3 | 22.3 | 29.6 | 25.5 |
| Ethanol | 649 | 51.9 | 15.8 | 8.8 | 19.4 | 26.5 | 21.3 |
| 1-Propanol | 649 | 50.7 | 16.0 | 6.8 | 17.4 | 24.6 | 18.7 |
| 1-Butanol | 651 | 49.7 | 16.0 | 5.7 | 15.8 | 23.2 | 16.8 |
| Methyl cyanide | 651 | 45.6 | 15.3 | 18.0 | 6.1 | 24.4 | 19.0 |
| DMSO | 652 | 45.1 | 18.4 | 16.4 | 10.2 | 26.7 | 19.3 |
| 1-Hexanol | 654 | 48.8 | 14.1 | 8.6 | 12.7 | 20.8 | 15.3 |
| DMF | 654 | 43.2 | 17.4 | 13.7 | 11.3 | 24.9 | 17.8 |
| Ethyl acetate | 656 | 38.1 | 15.8 | 5.3 | 7.2 | 18.2 | 8.9 |
| Acetone | 656 | 42.2 | 15.5 | 10.4 | 7.0 | 19.9 | 12.5 |
| CH_2_Cl_2_ | 657 | 40.7 | 18.2 | 6.3 | 6.1 | 20.2 | 8.8 |
| THF | 658 | 37.4 | 16.8 | 5.7 | 8.0 | 19.5 | 9.8 |
| Ether | 659 | 34.5 | 14.5 | 2.9 | 5.1 | 15.6 | 5.9 |
| CHCl_3_ | 661 | 39.1 | 17.8 | 3.1 | 5.7 | 19.0 | 6.5 |
| Hexane | 665 | 32.4 | 14.9 | 0.0 | 0.0 | 14.9 | 0.0 |

*The data of different solvent parameters in Table S1 is obtained from the literatures.^1-2^

1. **The determination of *K*, relevant data and parameters for analysis in different solvents**

**Figure S7.** The relationship of the absorbance and fluorescence peak intensity of **MC**.

By definition,^3^ the intensity of light absorbed by a compound in solvents (*I*_a_) with background absorption deduction is an exponential function of the corresponding absorbance of the compound (*A*):

|  | $I_{a}=I_{0}{(1-10}^{-A})$ | (1) |
| --- | --- | --- |

where *I*_0_ is the intensity of the incident beam and is a constant in this study. Since the fluorescence intensity of *F* varies linearly with the absorbed intensity *I*_a_,

|  | $F=kI_{a}$ | (2) |
| --- | --- | --- |

*F* also varies exponentially with absorbance:

|  | $F=kI_{0}{(1-10}^{-A})$ | (3) |
| --- | --- | --- |

where *k* can be approximately considered as fluorescence quantum yield. When the concentration of the compound is low, *F* may be proportional to *A*. The above formula could be approximately simplified into:

|  | $F=2.3kI_{0}A$ | (4) |
| --- | --- | --- |

As shown in Figure S7, the absorbance of **MC** at 529 nm instead of that at 365 nm in methanol has a good linear relationship with the fluorescence peak intensity of **MC** which meets the applicable conditions of formula 4. The concentration self-quenching effect of **MC** can be ignored below the experimental concentration. Thus, the concentration-independent value *K* can be obtained:

|  | $K=\frac{F}{A}=2.3kI_{0}$ | (5) |
| --- | --- | --- |

The calculated *K* values as well as other corresponding parameters of **MC** in different solvents are listed in the table below:

**Table S2** Values of different solvent parameters, *A*, *F* and *K* in different solvents

| Solvents | Viscosity  (25℃, mPa·s) | *δ*_T_ | *A* | *F* | *K* |
| --- | --- | --- | --- | --- | --- |
| Glycerol | 934.000 | 36.2 | 0.037 | 553919 | 14970771 |
| Ethylene Glycol | 16.100 | 33.0 | 0.301 | 908037 | 3016733 |
| 1-Hexanol | 4.580 | 20.8 | 1.026 | 1489530 | 1451784 |
| 1-Butanol | 2.540 | 23.2 | 1.062 | 1220750 | 1149482 |
| DMSO | 1.987 | 26.7 | 0.725 | 795578 | 1097349 |
| 1-Propanol | 1.945 | 24.6 | 1.017 | 1046910 | 1029410 |
| Ethanol | 1.074 | 26.5 | 1.334 | 964052 | 722677 |
| DMF | 0.794 | 24.9 | 1.170 | 689104 | 588978 |
| Methanol | 0.544 | 29.6 | 1.068 | 641625 | 600773 |
| CHCl_3_ | 0.537 | 19.0 | 1.101 | 604232 | 548803 |
| THF | 0.456 | 19.5 | 0.643 | 296843 | 461654 |
| Ethyl acetate | 0.423 | 18.2 | 0.506 | 267408 | 528474 |
| CH_2_Cl_2_ | 0.413 | 20.2 | 0.787 | 289282 | 367576 |
| Methyl cyanide | 0.369 | 24.4 | 1.036 | 193970 | 187230 |
| Acetone | 0.306 | 19.9 | 1.231 | 304041 | 246987 |
| Hexane | 0.300 | 14.9 | 0.352 | 52649 | 149570 |
| Ether | 0.224 | 15.6 | 0.366 | 144739 | 395462 |

*The data of different solvent parameters in Table S2 is obtained from the literatures.^4^

1. **Fluorescence emission behaviors of SP contained PEG with different molecular weight curing for 1h**

**Figure S8.** (a-f) Fluorescence emission spectra of **SP** contained PEG with different molecular weight; (a′-f′) irradiation time-dependent λ_max_ and their corresponding fluorescence intensities of **SP** contained PEG with different molecular weight.

1. **Fluorescence emission behaviors of SP contained PEG with different molecular weight curing for 7d**

**Figure S9.** (a-f) Fluorescence emission spectra of **SP** contained PEG with different molecular weight; (a′-f′) irradiation time-dependent λ_max_ and their corresponding fluorescence intensities of **SP** contained PEG with different molecular weight.

1. **OHV values of PEG with different molecular weights**

**Table S3** OHV values of PEG with different molecular weights

| PEG | PEG_2000_ | PEG_4000_ | PEG_6000_ | PEG_8000_ | PEG_10000_ | PEG_20000_ |
| --- | --- | --- | --- | --- | --- | --- |
| OHV | 57.00 | 29.00 | 18.75 | 14.00 | 11.15 | 5.65 |
| OHV^0.5^ | 7.54983 | 5.38516 | 4.33013 | 3.74166 | 3.33916 | 2.37697 |

**References**

1. Hansen, C. M., *Hansen Solubility Parameters: A User's Handbook, Second Edition*. CRC press: New York, 2007.

2. Reichardt, C.; Welton, T., *Solvents and solvent effects in organic chemistry*. John Wiley & Sons: 2011.

3. Fery-Forgues, S.; Lavabre, D., Are fluorescence quantum yields so tricky to measure? A demonstration using familiar stationery products. *J. Chem. Educ.* **1999,** *76* (9), 1260-1264.

4. Rumble, J., *CRC handbook of chemistry and physics*. CRC press: 2017.
